# Aluminum Doping Effect on Surface Structure of Silver Ultrathin Films

**DOI:** 10.3390/ma15020648

**Published:** 2022-01-15

**Authors:** Han Yan, Xiong Xu, Peng Li, Peijie He, Qing Peng, Can Ding

**Affiliations:** 1 School of Mechanical and Electronic Engineering, Wuhan University of Technology, Wuhan 430070, China; yanhan@whut.edu.cn (H.Y.); xu22002166@whut.edu.cn (X.X.); dcinwhut@whut.edu.cn (C.D.); 2 School of Power and Mechanical Engineering, Wuhan University, Wuhan 430072, China; hepeijie@whu.edu.cn; 3 Physics Department, King Fahd University of Petroleum & Minerals, Dhahran 31261, Saudi Arabia; 4 K.A. CARE Energy Research & Innovation Center at Dhahran, Dhahran 31261, Saudi Arabia; 5 Hydrogen and Energy Storage Center, King Fahd University of Petroleum and Minerals, Dhahran 31261, Saudi Arabia

**Keywords:** silver film, surface morphology, magnetron sputtering, molecular dynamics, first-principle

## Abstract

Ultrathin silver films with low loss in the visible and near-infrared spectrum range have been widely used in the fields of metamaterials and optoelectronics. In this study, Al-doped silver films were prepared by the magnetron sputtering method and were characterized by surface morphology, electrical conductivity, and light transmittance analyses. Molecular dynamics simulations and first-principles density functional theory calculations were applied to study the surface morphologies and migration pathway for the formation mechanisms in Al-doped silver films. The results indicate that the migration barrier of silver on a pristine silver surface is commonly lower than that of an Al-doped surface, revealing that the aluminum atoms in the doping site decrease the surface mobility and are conducive to the formation of small islands of silver. When the islands are dense, they coalesce into a single layer, leading to a smoother surface. This might be the reason for the observably lower 3D growth mode of silver on an Al-doped silver surface. Our results with electronic structure insights on the mechanism of the Al dopants on surface morphologies might benefit the quality control of the silver thin films.

## 1. Introduction

Ultrathin metallic films are used for innovative applications, such as flexibility transparent electrodes [1,2], optical metamaterial lenses [3], plasmonic devices [4,5], and light-emitting diodes [6,7]. Due to their advantages in electrical conductivity and low loss in the visible to near-infrared spectrum, ultrathin silver films have attracted a great deal of interest [8]. However, a rough surface with large grain size severely affects the film conductivity and results in additional optical loss. Using conventional deposition techniques for fabricating silver films, including electron beam evaporation and magnetron sputtering, it is difficult to obtain ultrathin and smooth silver film on dielectric substrate surfaces due to the Volmer Weber growth mode during the film deposition process [9].

To address the issue of the roughness of ultrathin silver films, numerous approaches have been applied. A widely used method is to deposit a seed layer before the silver film. Ciesielski et al. investigated the crystallinity and optical parameters of silver layers of 10–35 nm thickness using a 2–10 nm-thick germanium wetting layer pre-deposited on fused silica substrates [10]. Remy et al. prepared 15 nm silver thin films using a transparent aluminum nitride seed layer in an argon and nitrogen atmosphere. They found that the percolation threshold of silver thin films was reduced by aluminum nitride seeding [11]. Some researchers have provided critical insights into the development of noninvasive growth manipulation strategies, in which surface morphology can be promoted by deploying a gaseous species atmosphere. Pliatsikas et al. studied the morphological evolution of magnetron-sputtered silver thin films deposited on weakly interacting silicon dioxide substrates using oxygen as a surfactant. They found that the presence of oxygen throughout all film formation stages leads to smoother film surfaces and larger electrical resistivity of the silver layer [12]. The presence of nitrogen in the gas atmosphere reported by Jamnig et al. revealed similar results [13]. Another improvement in surface roughness was achieved by the co-doping method during silver deposition. Zhang et al. obtained ultrathin and smooth silver films by doping a small amount of aluminum during film deposition, and they found that the thermal stability of Al-doped silver film is significantly improved compared to pure silver [14]. Thomas et al. implemented ultra-thin metallic films in dielectric/metal/dielectric structures using aluminum-doped zinc oxide as the dielectric and an ultrathin aluminum doping silver as the metallic interlayer [15]. In addition to these experiments, there are also many theoretical studies on the surface adsorption of silver [16,17,18,19]. Although previous studies have shown that the kinetic energy of the aluminum dopant atom slightly affects the surface morphology of silver films [20], the atomic-scale mechanisms that drive the morphology formation and the evolution of Al-doped silver films are elusive.

In this paper, the roles of aluminum doping in silver film are studied by experiment and first-principles calculations. The surface morphologies for the layer-by-layer growth of silver on an amorphous silicon dioxide surface with aluminum as a dopant are studied by molecular dynamics (MD) simulations. The surface migration behavior of both silver and aluminum on pristine and Al-doped silver surfaces is investigated using first-principles calculations with the frame of Density Functional Theory (DFT). To understand the stabilities of adsorption models, the adsorption energies of silver and aluminum adatoms with different migration paths are systematically examined.

## 2. Methods

### 2.1. Experiment Methods

The samples were prepared using magnetron sputtering equipment (MSP-3200, Beijing Chuangshiweina Technology Co., Ltd., Beijing, China). Polished silica glass substrates were used for the deposition of silver films. The substrates were cleaned using ultrasonic deionized water and then dried using nitrogen gas. The chamber was pumped to an initial pressure of 3 × 10^−3^ Pa before the deposition process. The rotating speed of the substrate tray was set to be 20 rpm/min, and no deliberate heating was applied to the substrate during the whole deposition. The metallic films were prepared by the sputtering of silver and aluminum targets at room temperature. The chamber was cooling by circulating water (25 °C). The substrate was attached to a stainless-steel holder with good thermal conduction, and the temperature rise of substrate can be neglected [21,22]. The purity of the sputtering materials was 4 N for silver and aluminum. The diameter and thickness of the sputtering targets are 75 mm and 5 mm, respectively. The distance between the sputtering target and substrate was kept at 35 cm. The magnetron sputtering sources were tilted in relation to the axis of the substrate holder. All the depositions were performed in an atmosphere of argon, and the deposition pressure was set as 0.6 Pa for each film growth process. The silver target was sputtered by radio frequency (RF) power, while the aluminum target was sputtered by direct current (DC) power. The frequency of the RF power supply was 13.56 MHz. Based on previous experimental results, when both RF and DC power were set to be 200 W, silver and aluminum were sputtered at an average deposition rate of approximately 6 Å/s and 0.6 Å/s, respectively. The surface morphologies of the samples were tested by atomic force microscopy (AFM, Veeco NanoScope MultiMode, Bruker Co., Ltd., Madison, WI, USA), and Nanoscope 7.30 software (Bruker Co., Ltd., Madison, WI, USA) was used for image analysis. The room temperature sheet resistances measurements of the samples were carried out by a four-point probe system (ST-2258C, Suzhou Jingge Electronic Co., Ltd., No.18 Xinmen Road, Suzhou, China ). The transmittance spectra of the samples were measured by UV/VIS/NIR Spectrometer (Lambda 750S, PerkinElmer Inc., Waltham, MA, USA), which is defined as the difference in transmittance between silica substrate with and without silver film.

### 2.2. Molecular Dynamics Simulations

Although the formation and evolution of the rough morphology of metallic film on an oxidized substrate were understood from a thermodynamic point of view [23,24], molecular dynamics simulation can analyze the influencing factors of the surface topography from a different perspective. The effect of aluminum doping on the surface morphology of silver films on amorphous silicon oxide substrate was studied using Large-scale Atomic/Molecular Massively Parallel Simulator (LAMMPS) code [25,26]. The simulation box consisted of a substrate region and deposited film region, and the dimensions were 114.04 Å (*x*-axis) × 85.53 Å (*y*-axis) × 120.00 Å (*z*-axis). The substrate region was filled with amorphous silicon dioxide; that is, the atoms in the substrate were arranged in a disorderly manner, corresponding to the silica glass substrate in the macro system. The deposition atoms of silver and aluminum were injected from the top of deposited film region. The Tersoff potential [27], embedded-atom method (EAM) potential [28], and Lennard Jones (LJ) potential [29] were used for the Si-O system, metallic atoms, and dynamic interactions between atoms, respectively. The incident direction of deposition atoms in each calculation model was perpendicular to the substrate surface. The initial incidence velocity of each deposition atom was set to 6 Å/ps.

### 2.3. First-Principles Calculations

The adsorption and migration pathway of deposition of a pre-deposited silver surface were studied by first-principles calculations within the frame of density functional theory. The Quantum Espresso package [30,31], version 6.8, was used for the calculations, which has been widely used for the study of atom adsorption and migration [32]. In our simulations, the interactions between ionic cores and valence electrons were described by the projector augmented wave (PAW) potentials [33]. The exchange functional and correlation energy were expressed by Perdew–Burke–Ernzerhof (PBE) [34]. The electronic wave functions were expanded with an energy cutoff of 60 Rydberg. The total energy of each calculation was converged to 1 × 10^−5^ eV. The nonlocal van der Waals interactions corrections are considered [35].

**Figure 1:**
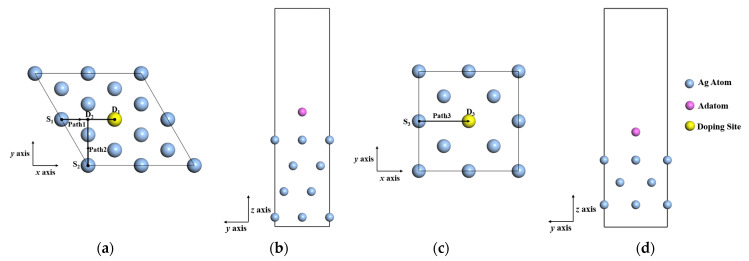
Schematic illustration of adatom migration on silver surface. (**a**) Top view of migration paths on Ag (111) surface, and D_1_ denotes the doping site; (**b**) side view of adsorption distance between adatom and Ag (111) substrate; (**c**) top view of migration paths on Ag (100) surface, and D_3_ denotes the doping site; (**d**) side view of adsorption distance between adatom and Ag (100) substrate.

Slab models consisting of a substrate layer and vacuum layer were used for simulations, as shown in Figure 1. To investigate the effect of aluminum doping on the deposition of silver film, a single silver atom is substituted by one aluminum atom to denote the aluminum doping silver surface. To understand the aluminum doping mechanism including different lattice planes, the Ag (111) and Ag (100) surface were investigated.

Three series of paths were considered to reveal the effects of aluminum doping on the preferred adsorption configuration and migration of adatoms. Path 1 represents the migration of adatom from S_1_ to D_1_, as shown in Figure 1a. Path 2 represents the migration of adatom from S_2_ to D_2_. Path 3 represents the migration of adatom from S_3_ to D_3_, as shown in Figure 1c. The adsorption distance was defined as the nearest distance between the adsorbed atom and surface plane, as shown in Figure 1b,d. On each adsorption site, the initial adsorption distance of adatoms above the silver surface was set as 2.5 Å. Geometry optimizations were performed for different adsorption sites, in which the bottom substrate layers were fixed at lattice positions, while positions of the adatoms were allowed to relax along the z-axis. The optimized position of the adatom was calculated by minimizing the total energy of the system using the Broyden–Fletcher–Goldfarb–Shanno (BFGS) method [36]. Periodic boundary conditions were applied along the supercell axial directions. The lattice constant of silver unit cell was obtained by lattice optimization, which was 4.0857 Å—slightly lower than the reference value of 4.09 Å [37].

The adsorption energy *E*_ad_ of an adsorbed atom on silver surface is defined as follows [38]:

*E*_ad_ = *E*_total_ − *E*_adatom_ − *E*_substrate_ (1)

where *E*_total_ is the total energy of the substrate with the adsorbed atom, *E*_surface_ stands for the total energy of the substrate, and *E*_adatom_ is the total energy of an isolated adsorbed atom.

## 3. Results and Discussion

### 3.1. Experimental Samples Characterization

Silver thin films with different aluminum components were prepared by magnetron sputtering equipment. The sputtering power of the silver target was kept as constant, while the sputtering power of the aluminum target and total deposition time varied. The deposition parameters are listed in Table 1.

**Figure 2:**
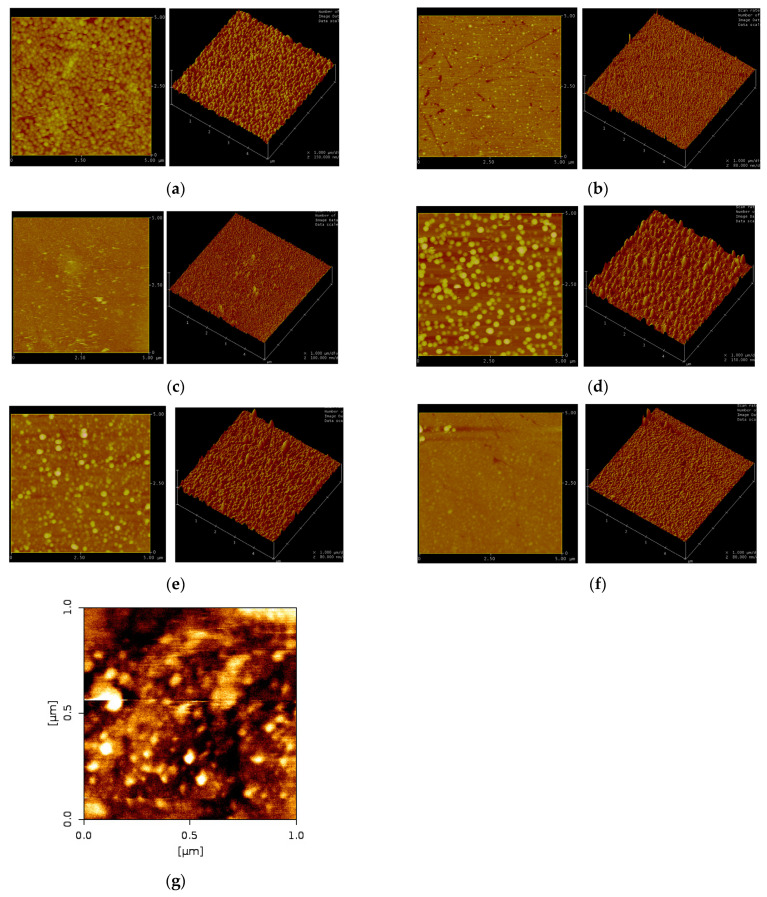
AFM images of each sample. (**a**) Sample 1, (**b**) sample 2, (**c**) sample 3, (**d**) sample 4, (**e**) sample 5, (**f**) sample 6, (**g**) substrate. The scan parameters are: scan size is 5.000 μm, scan rate is 1.001 Hz, number of samples is 256, image date is height, x is 1.000 μm/div and z is varied from 80 to 150 nm/div.

**Figure 3:**
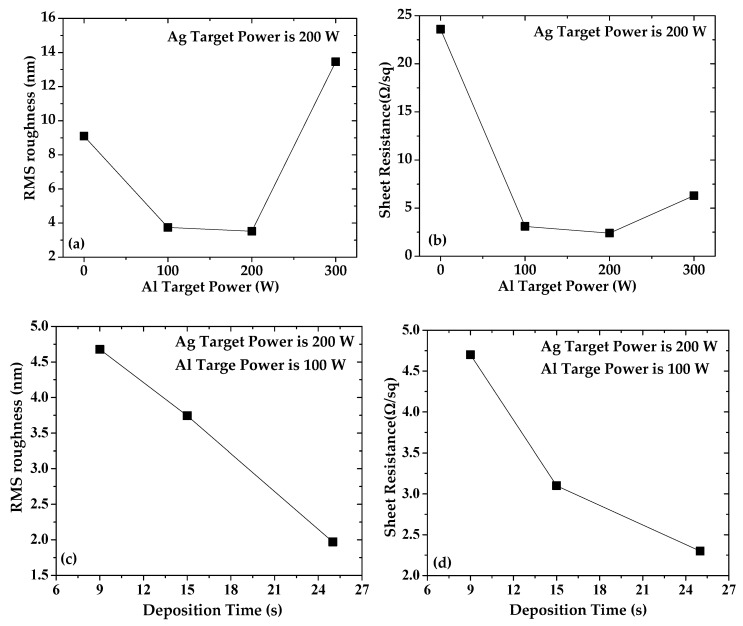
The RMS roughness (**a**) and sheet resistance (**b**) of Al-doped silver films under different Al target power. The RMS roughness (**c**) and sheet resistance (**d**) of Al-doped silver films under different deposition time.

The results of the surface morphologies of the samples are shown in Figure 2. The RMS value of silica substrate is 0.726 nm. The undoped silver film shown in Figure 2a has a rough surface and many large protrusions, and therefore, the RMS value of undoped silver film is as large as 9.096 nm. The RMS value of Al-doped silver film is 3.745 nm, which is significantly smaller than that of the undoped silver film. It is obvious that doping silver film with a small amount of aluminum atoms can reduce the surface roughness effectively and promote the formation of a smoother silver film. The surface morphologies of silver films with different aluminum compositions are shown in Figure 2b–d. It can be seen that the RMS values of the Al-doped silver film with 100 W, 200 W, and 300 W aluminum target sputtering power are 3.745 nm, 3.523 nm, and 13.456 nm, respectively. The RMS results are summarized in Figure 3a. It can be seen that the surface roughness can be further improved by increasing the doping concentration of aluminum atoms. However, the surface morphology deteriorates when the doping concentration increases significantly.

The room temperature sheet resistances of silver films were measured using a four-probe meter. Sample 1 was undoped silver film, and its sheet resistance was 23.6 Ω/sq. Sample 2 was Al-doped silver film with 100 W aluminum target sputtering power, and its sheet resistance was 3.1 Ω/sq, which is much lower than that of undoped silver film. It is indicated that the degree of surface smoothness has a positive effect on sheet resistance, and that doping a small amount of aluminum atoms not only helps to form smooth silver films but also makes its sheet resistance smaller. The sheet resistance of the Al-doped silver film with aluminum sputtering power of 100 W, 200 W, and 300 W is 3.1 Ω/sq, 2.4 Ω/sq, and 6.3 Ω/sq, respectively. The sheet resistance of Al-doped silver film as a function of aluminum target power is shown in Figure 3b. The results show first a decrease and then an increase with an increase in aluminum composition, which corresponds to the degree of surface smoothness. They reveal that a small amount of aluminum atoms promotes the sheet resistance of Al-doped silver film, and excessive aluminum atoms decrease the sheet resistance.

The surface morphologies of Al-doped silver film with different deposition times were also analyzed. The sputtering powers of silver and aluminum targets were kept constant while the deposition times were set to 9 s, 15 s, and 25 s, respectively. The corresponding AFM results are shown in Figure 2b,e,f. The size of the surface protrusions gradually decrease as the thickness of Al-doped silver film increases. The surface morphology of the Al-doped silver film will become smoother as its thickness increases. The sheet resistance of the Al-doped silver film with deposition times of 9 s, 15 s, and 25 s was 4.7 Ω/sq, 3.1 Ω/sq, and 2.3 Ω/sq, respectively. Figure 3d shows the sheet resistance of Al-doped silver film as a function of deposition time. It was indicated that the sheet resistance decreases with an increase in thickness.

**Figure 4:**
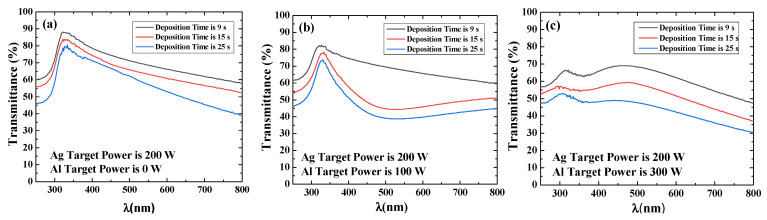
Transmittance results of undoped silver film and Al-doped silver film with different deposition time, and the aluminum target power is (**a**) 0 W, (**b**) 100 W and (**c**) 300 W, respectively.

The transmittance spectra of the Al-doped silver films are shown in Figure 4. It is found that the transmittance of both undoped silver films and Al-doped silver films decreases with the increase in deposition time. The significant drop in transmittance spectra is caused by aluminum doping. The localized plasmon absorption of metal nanoparticles would explain the tendency toward comparatively low optical transmittance [39,40]. That is, aluminum doping can affect the particle size and nuclear density of silver nanofilm during deposition. Therefore, the resistivity and transmittance of silver thin films need to be considered together in the application of transparent electrodes.

### 3.2. MD Simulations on Atomic Structures

**Figure 5:**
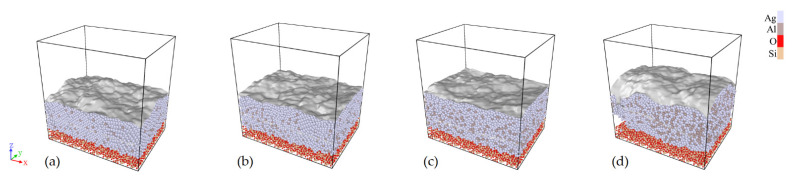
Surface roughness of Al-doped silver films by molecular dynamics simulations. The number of aluminum atoms was set to be (**a**) 1000, (**b**) 2000, (**c**) 4000, and (**d**) 8000, while the number of silver atoms was kept at 20,000.

The atomic-scale deposition of aluminum doped silver films on amorphous silicon dioxide substrate with different aluminum compositions and different deposition time were studied by molecular dynamics simulations. The open visualization tool (OVITO) [41] was used to visualize and calculate the surface roughness by root-mean-square (RMS); the results were represented with the Gaussian density method [42] and shown in Figure 5. In order to simulate the effect of aluminum doping on the surface morphology of Al-doped silver film, 20,000 silver atoms were deposited in the system; the number of aluminum atoms varied from 1000 to 8000 to change the doping composition. Figure 5a–d show the simulation results of deposition dynamics with varied aluminum composition. The RMS value of Al-doped silver film with an aluminum composition of 5% and 10% is 3.296 Å and 2.670 Å, respectively. The surface roughness decreases with the increase in aluminum compositions, which is similar to the outcome of varying aluminum target power from 100 to 200 W. The RMS value of Al-doped silver film with aluminum composition of 20% and 40% is 3.081 Å and 8.028 Å, respectively. The results indicate that excessive aluminum doping increases the surface roughness of Al-doped silver films, which is consistent with the tendency toward variation of our experimental results.

**Figure 6:**
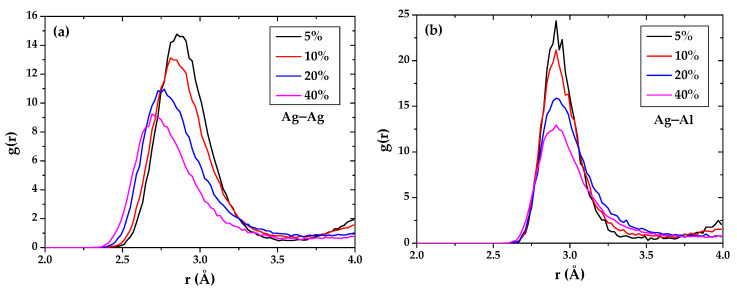
The radial distribution function of (**a**) Ag–Ag and (**b**) Ag–Al in Al-doped silver film deposited in different aluminum composition.

The radial distribution function (RDF) of the Ag–Ag bond and Ag–Al bond of the Al-doped silver film deposited in different aluminum composition are calculated by OVITO and shown in Figure 6. The peak of RDF of the Ag–Ag bond that occurs in 2.85 Å with aluminum doping is 5%, which is smaller than the length of the silver bond of 2.889 Å in reference [43]. It is found that the atomic bonds of Ag–Ag in 5% Al-doped silver film are distributed from 2.39 to about 3.5 Å for the nearest neighbors. The minimal pair distance of Ag–Ag is reduced to 2.29 Å when the aluminum composition increases to 40%, and the peak position of the Ag–Ag pair distance also decreases as the composition of aluminum increases. The results reveal that the incorporation of aluminum makes the nearest neighbor atoms of the silver atoms in the silver film smaller. Figure 6b shows that the pair distance of Ag–Al remains no matter whether the aluminum composition changes. In our opinion, the nearest neighbor distance of silver–aluminum is almost unaffected by the number of aluminum atoms.

### 3.3. First-Principles on Adsorptions and Migration

**Figure 7:**
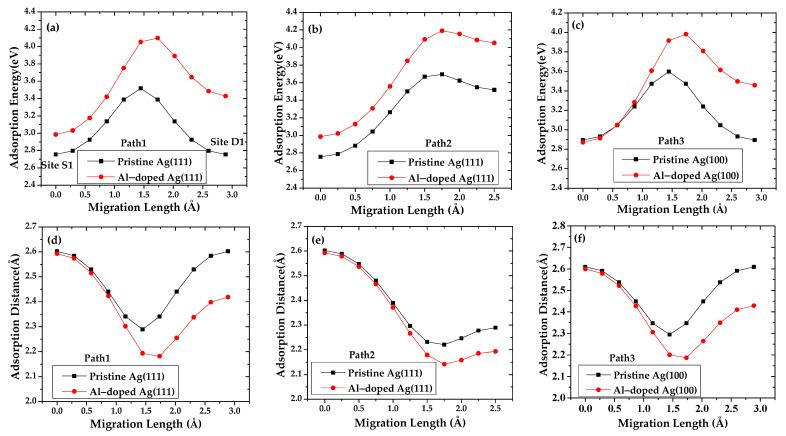
Adsorption energy of silver adatom migration pristine Ag (111)/Ag (100) surface and Al-doped Ag (111)/Ag (100) surface along (**a**) path 1, (**b**) path 2 and (**c**) path 3, and corresponding adsorption distances of silver adatom migration are represented by (**d**) path 1, (**e**) path 2 and (**f**) path 3.

To obtain a more detailed description of the atomic origin of aluminum doping effects on the initial deposition on Al-doped silver films, first-principles computational studies of the adsorption and migration of aluminum and silver adatom on a pristine silver and an Al-doped silver surface are presented. The absolute adsorption energies for silver adatom on a pristine Ag (111) and an Al-doped Ag (111) surface are shown in Figure 7. The relatively larger value in absolute adsorption energy indicates the preference of adatom adsorption on the surface.

The results show that the adsorption energy of silver on the Al-doped Ag (111) surface along path 1 and path 2 is higher than that of the pristine Ag (111) surface. Therefore, the silver atoms deposited on the Al-doped Ag (111) surface are more stable. The preferred adsorption position for silver on a pristine Ag (111) surface along path 1 is the D_2_ site, which is located in the middle of the bridge between the S_1_ site and D_1_ site, and it is close to the hollow site above the three-fold silver atoms. The maximal adsorption energy and corresponding adsorption distance of silver along path 1 are 3.518 eV and 2.289 Å, respectively. In the case that the silver atom at D_1_ is replaced by an aluminum atom, the maximum adsorption energy and minimum adsorption distance of silver that migrate along path 1 are not at the D_2_ site but are closer to the D_1_ site. The corresponding energy and distance are 4.098 eV and 2.182 Å, respectively. The calculation results show that the silver adatoms tend to be adsorbed at the site near the aluminum doping atom, the adsorption distance is smaller, and the bond between the adatom and the surface is stronger. For the same reason, the subsequently deposited silver atoms can also be adsorbed in the vicinity of the aluminum atom. In the case that silver migrates along another pathway perpendicular to path 1, the energetic adsorption position appears in the hollow site, and the corresponding adsorption energy on the pristine Ag (111) surface is 3.694 eV, which is larger than that in the case of path 1. From the standpoint of the minimum energy of the system, the silver adatom on top of the silver substrate atom is considered to be unstable because it moves toward the bridge site, close to the hollow site. From the crystallographic point of view, the initial stacking growth sequence of the Ag (111) layer continues the FCC stacking of the substrate [44,45]; hence, the best adsorption site in the second layer is the hollow position of the first layer.

The maximal adsorption energy along path 2 increased to 4.191 eV with aluminum doping, which revealed that aluminum doping improves the formation of silver adatom deposition in the FCC-stacked sequence. It can be seen from Figure 7c that as the silver atom approaches the aluminum doped atom, the difference between the adsorption energy of the silver adsorbed on the Al-doped Ag (111) surface and pristine Ag (111) surface gradually increases, indicating that the aluminum doping site induces a more stable interaction with the silver adatom. The maximal adsorption energies of silver adsorption on pristine Ag (100) and Al-doped Ag (100) along path3 are 3.597 eV and 3.982 eV, respectively. The corresponding adsorption distances of silver on pristine Ag (100) and Al-doped Ag (100) are 2.294 and 2.187 Å, respectively. The aluminum-doped atom in the (111) plane has a greater influence on silver atoms than that in the (100) plane.

According to the transition-state theory [46,47], the migration barrier can be defined as the difference between the maximum saddle adsorption energy and minimum valley adsorption energy in the migration path. In our calculations, the migration barriers of silver adsorption on pristine Ag (111) along path1 is 0.762 eV, which is relatively small in comparison with that along path 2 of 0.939 eV. The migration barriers of silver adsorption on Al-doped Ag (111) along path 1 and path 2 are 1.113 eV and 1.206 eV, respectively. The stronger binding of silver to the Al-doped surface is also reflected in the finding that the migration barrier of silver on pristine Ag (111) is commonly lower than that of the Al-doped surface. As a consequence, the aluminum atoms in the doping configurations decrease the surface mobility of silver by increasing the diffusion barrier for silver adatoms approaching the aluminum center. Therefore, the presence of substitutional aluminum is conducive to the formation of small islands of silver. If the islands are dense, one expects that they coalesce into a single layer. This might be the reason for the lower 3D growth mode of silver on an Al-doped Ag (111) surface. The results of silver adsorption on an Ag (111) surface are in agreement with the above standpoint. The migration barrier of silver adsorption on pristine Ag (100) and Al-doped Ag (100) along path 3 is 0.703 eV and 1.110 eV, respectively. The calculation results reveal that the presence of aluminum-doped atoms significantly increases the migration barrier of silver atoms for path 3 of the Ag (100) surface.

**Figure 8:**
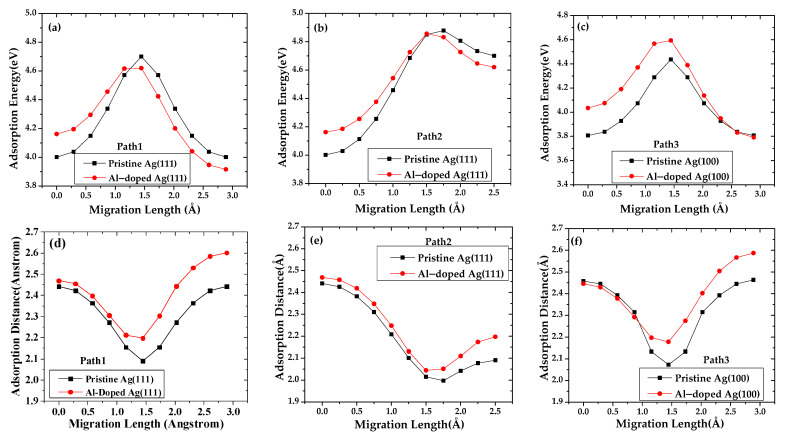
Adsorption energy of aluminum adatom migration pristine Ag (111)/Ag (100) surface and Al-doped Ag (111)/Ag (100) surface along (**a**) path 1, (**b**) path 2 and (**c**) path 3, and corresponding adsorption distances of aluminum adatom migration are represented by (**d**) path 1, (**e**) path 2 and (**f**) path 3.

The Al-doped surface not only affects the adsorption and migration of the silver adatom but also impacts the subsequent deposition of dopant atoms. The calculation results shown in Figure 8 reveal that the surface migration of aluminum adatoms is different from that of silver adatoms. The maximal adsorption energy of aluminum that migrates along path 1 on pristine Ag (111) and Al-doped Ag (111) is 4.701 eV and 4.620 eV, respectively. The corresponding minimum adsorption distance is increased from 2.091 to 2.197 Å. In the case that aluminum migrates along path 2, the maximal adsorption energy of aluminum on pristine Ag (111) and Al-doped Ag (111) is 4.879 eV and 4.856 eV, respectively. The corresponding minimum adsorption distance is increased from 1.997 to 2.044 Å. It is found that the adsorption energy of the Al adatom at site S_1_ on a pristine Ag (111) surface is smaller than that on an Al-doped Ag (111) surface, as the aluminum atoms migrate along path 1 and path 2, and the adsorption energy of the aluminum adatom at the D_1_ site of pristine Ag (111) becomes lower than that of Al-doped Ag (111) due to aluminum doping.

We also consider the adsorption pathway on Ag (100) surfaces. The maximal adsorption energy of aluminum adsorption on pristine Ag (100) and Al-doped Ag (100) is 4.436 eV and 4.594 eV, respectively. The corresponding adsorption distance of aluminum on pristine Ag (100) and Al-doped Ag (100) is 2.073 and 2.177 Å, respectively. Aluminum doping increases the adsorption energy of aluminum on the Ag (100) plane, which suggests that aluminum atoms are more likely to grow epitaxially along another direction. The migration barriers of aluminum adatom on pristine Ag (111) and Al-doped Ag (111) along path 1 are 0.699 eV and 0.704 eV, respectively. However, the migration barriers of aluminum adatom on pristine Ag (111) and Al-doped Ag (111) along path 2 are 0.877 eV and 0.694 eV, respectively. The calculation results reveal that the presence of aluminum-doped atoms slightly increases the migration barrier of aluminum atoms for path 1, while it greatly decreases the migration barrier of aluminum atoms along path 2. In the case of the Ag (100) plane, the corresponding value on pristine and Al-doped surface is 0.629 eV and 0.823 eV, respectively. Aluminum doping significantly reduces the migration length of aluminum atoms on the (100) plane of the silver layer.

In a word, the adsorption energy of the aluminum adatom is larger than that of the silver atom at the same adsorption site on the silver surface. The corresponding adsorption distances of aluminum adatoms are lower than those of silver adatoms. It is difficult for silver atoms to move on the surface of silver doped with aluminum, and the surface migration and diffusion length is short. This further explains the fewer three-dimensional silver growth patterns observed on the Al-doped silver surface.

### 3.4. Electronic Structures

**Figure 9:**
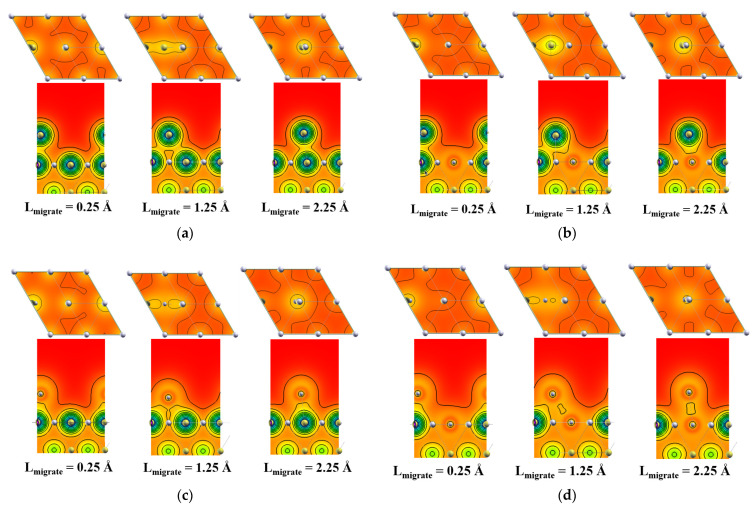
Top and side view of charge density potential energy isolines of adatom adsorption and migration on the Ag (111) surface along path 1. (**a**) Silver adatom migration on the pristine Ag (111) surface, (**b**) silver adatom migration on the Al-doped Ag (111) surface, (**c**) aluminum adatom migration on the pristine Ag (111) surface, (**d**) aluminum adatom migration on the Al-doped Ag (111) surface.

Charge density can reveal the charge transition and bonding polarization direction between adatom and surface atoms [48,49]. The results are represented by Xcrysden [50,51], as shown in Figure 9. The side view of the iso-surface of charge density mapping projected on the parallel x–z plane containing adatoms along path 1 and the top view of the slice surface between adatoms and the Ag (111) surface are shown. It should be noted that there is an obvious interaction between adatoms and the outermost surface atoms of Ag (111), while the interaction with the second or inner layer is relatively small.

Compared with the pristine silver interface, the electronic accumulation degree increases around the silver adatom, resulting in improved ionicity nearby. However, the overlap degree of an electron cloud at the interface of aluminum adatom with the doping atoms decreases. The aluminum atoms at the doping position greatly affect the charge distribution between the adsorbed atoms and the substrate.

## 4. Conclusions

In this work, the impact of aluminum doping on the surface formation mechanism was studied. The results of fabricating silver nanofilms through an aluminum co-sputtering process revealed that a small amount of aluminum reduces the surface roughness and promotes the sheet resistance of Al-doped silver film, while excessive aluminum deteriorates the surface roughness and decreases the sheet resistances. The theoretical calculation results show that the adsorption energy of silver adatoms on the Al-doped silver surface is higher than that of the pristine silver surface, and silver adatoms tend to be adsorbed at the site near the aluminum doping atom. The aluminum atoms in the doping configurations decrease the surface mobility of silver by increasing the diffusion barrier for silver adatoms approaching the aluminum center. Aluminum doping atoms provide energetically favorable nucleation sites for the incoming silver atoms, which enhances the nuclei density of the silver layer on the surface, leading to an improved surface morphology. Our results might be helpful in surface quality control of the ultrathin silver films.

## Figures and Tables

**Table 1:** Process parameters for each sample.

Sample No.	Deposition Time (s)	Sputtering Power of Ag (W)	Sputtering Power of Al (W)
1	15	200	0
2	15	200	100
3	15	200	200
4	15	200	300
5	9	200	100
6	25	200	100

## Data Availability

The data presented in this study are available on request from the first author and corresponding author.
